# Appendicitis in Relation to Disease of the Uterine Adnexæ and Pregnancy1Read at the annual meeting of the American Association of Obstetricians and Gynecologists, Niagara Falls, August 17-20, 1897.

**Published:** 1897-11

**Authors:** John B. Deaver

**Affiliations:** Philadelphia, Pa., Surgeon-in-chief of the German Hospital


### APPENDICITIS IN RELATION TO DISEASE OF THE UTERINE ADNEXÆ AND PREGNANCY.[1]

BY JOHN B. DEAVER, M. D., Philadelphia, Pa.,
Surgeon-in-chief of the German Hospital.

A PAPER upon the subject of appendicitis, with its fatality and many complications and the close relationship which it may bear to contiguous organs and structures, will be of particular interest to this body of obstetricians and gynecologists. You will,

1. Read at the annual meeting of the American Association of Obstetricians and Gynecologists, Niagara Falls, August 17-20, 1897.

no doubt, agree with me that acute appendicitis is the most common and one of the most fatal of all acute intraäbdominal affections. Furthermore, that this mortality is in a great measure due to a failure to make an early diagnosis and to the character of the treatment instituted. The position which I have held—namely, to operate as soon as the diagnosis is made—I still maintain. Assuming that this is done very shortly after the onset of the attack, I know that this course is attended by the least mortality. My experience in the operative treatment of this dangerous, treacherous and fatal disease embraces many hundred cases, and I am prepared to make the statement that I have never seen a human life lost where operation was performed immediately following the onset of the disease. While I am aware that to discriminate between cases and to be able to say from the onset which are operative cases and which will recover without operation, or to be able to defer operation with absolute safety for two, three or more days rather than to rush in, as some put it, and remove the diseased appendix as soon as the diagnosis is made, may be more scientific, yet I know positively that this course is at the cost of many human lives. When, therefore, it becomes a question of following the lines of theory or that of experience which teaches practical facts and which when instituted will bring practical and favorable results, to my mind permits of no choice. The ultimate results of inflammation of this organ more than justify early operative interference.

Let us consider for a moment some of the results of appendiceal inflammation. First and most important, because of the high rate of mortality, is perforation of the appendix with general septic peritonitis. Next, perforation with localised abscess formation which, if not evacuated early, produces general sepsis, a tedious convalescence, a granulating wound and in many instances ventral hernia or fecal fistula. Appendicitis followed by large localised abscess necessarily means the destruction of a great deal of tissue which in some instances nature fails to repair, to say nothing of the loss of life when it occurs in the young or feeble. I consider the formation of a localised abscess occurring as the result of an appendicitis anything but the most fortunate culmination of the attack for the following reasons—namely, because the inflammation should not be allowed to reach that stage ; that in the hands of the majority of operators it is an excuse to leave the appendix, believing that with the evacuation of the abscess they have accomplished all that can be expected. This practice not only offers an excuse in too many instances for leaving behind an appendix which is more liable to be the cause of subsequent and less favorable attacks, but also exposes the patient to the risk of an undiscovered secondary collection which in my experience is a frequent cause of death.

Before a body of operators comprising the membership of this association it would be presumptuous for me to draw a comparison between operation in the absence of and in the presence of pus. I may, however, bring to your attention some complications of pus formation which I have met with.

The presence of pus increases the danger of lymphatic infection. This is also true of infection through the portal system, (pyelo-phlebitis,) resulting in miliary abscess of the liver and death. The complications which may arise from an undisturbed localised appendiceal abscess are its rupture into the bladder, thus occasioning an entero-vesical fistula ; rupture into a bronchus and evacuation by way of the mouth ; rupture into the ascending colon, rectum or vagina, which is not to be considered a favorable termination and does not preclude the possibility of a recurrence of the inflammation; rupture into the ureter with evacuation of pus through the bladder. A complication which is not an uncommon sequence of the formation of a localised abscess is intestinal obstruction, due to adhesions which have been formed in the process of localisation. Again, secondary obstruction may follow the evacuation of an abscess, due to the subsequent contraction of its walls. A septic phlebitis, phlegmasia alba dolens, is not an uncommon sequence of localised abscess formation.

A condition of affairs which may arise as the result of a small collection which has remained undisturbed, is when the abscess wall has undergone a cheesy degeneration, involving in the process contiguous structures. When the bowel has been thus included an attempt to remove the degenerated mass will frequently bring to light a perforation of the bowel, often so large as to necessitate excision, enterorrhaphy or an anastomosis. In operating for appendicitis, the multiplication of the difficulties induced by pus formation must prepare the surgeon to expect to meet and handle any of the complications of abdominal surgery.

To recapitulate, the large mortality in appendicitis, as I have previously stated, is the result of too conservative measures in the early stage of the disease. Surgeons whose experience in the treatment of appendicitis has been large, I am sure, will to a man agree with me in this statement. To be brief, I have yet to see a human life lost where, in an acute attack of appendicitis, the appendix has been removed within six to eight hours after the onset of the disease, while, on the other hand, I regret to say that I have seen many human lives lost by adopting the so-called conservative methods—namely, opium to relieve the pain, allowing the bowels to remain confined, leeches, fly blisters, turpentine stoops applied—any or all of which are not only useless but harmful. I cannot make this too strong, as I have seen five deaths in as many days, in all of which cases I am morally certain that had the appendix been removed immediately after the onset of the first pain all would have recovered.

The question naturally arises, who is largely responsible for this high mortality ? Is it our teachers ? Personally I feel that it is. What is the alternative ? I would suggest that our professors in the different teaching institutions witness a number of operations done early (most of them see them done late), when I know there will no longer be the list of sceptics there are now. This, too, would be a healthful diet for the so-called surgical critic who writes articles condemning too frequent operation. The last-mentioned class evidently are wanting in experience, otherwise they could not but entertain different views.

The conditions in the female which call for careful thought in differentiating between one or other of the diseases of the uterine adnexæ and acute or chronic appendicitis are the following :

1. *Acute Salpingitis.*—There is frequently a close relationship between acute catarrhal appendicitis and right-sided acute salpingitis, due to Clado's ligament and the symptoms may as a result closely resemble each other. The great points of difference, however, are the history of infection, the slight degree of abdominal tenderness and rigidity, the location of the pain and the absence of vomiting, and in the case of appendicitis the pain migrating from epigastric or periumbilical to the right iliac fossa, the decided tenderness and rigidity of the lower right quadrant of the abdominal wall, suddenness of attack, accompanied by vomiting and no history of infection, the absence of signs by vaginal examination and the presence of a palpably enlarged appendix.

2. *Pyosalpinx* and *Ovarian Abscess.*—The presence in the recto-uterine cul-de-sac of an inflammatory mass in intimate relation with the uterus, which renders it partially or completely immovable and which can be clearly outlined by vaginal, bimanual or combined vaginal and rectal examination, together with the history of a vagino-uterine infection and the presence of a septic fever, established the diagnosis of pyosalpinx or ovarian abscess. The essential points in the differentiation between these two affections and appendicitis are the suddenness of onset in the latter, accompanied by vomiting, the characteristic rigidity and tenderness following general abdominal pain.

Inflammation of the right ovary may be confounded with appendicitis, as it is attended with pain, tenderness in the right iliac fossa, nausea and fever. It is, however, always accompanied by disturbances of the uterine functions, is accompanied by the characteristic "ovarian" pain and is demonstrable by vaginal or bimanual examination. The tenderness is never so intense as in appendicitis and is not accompanied by a perceptibly enlarged appendix.

3. *Extrauterine Pregnancy.*—The history in these cases is usually that of partial or complete cessation of the menstrual flow for one, two or more periods, generally accompanied by other symptoms of pregnancy, with collapse supervening upon an attack of acute abdominal pain. The pain is long-continued and paroxysmal, but not colicky. An irregular, bloody, vaginal discharge, generally lighter in color than the normal menstrual flow and containing shreds of tissue, portions of the decidua, is present. Vaginal examination will detect a tender and sensitive mass in the posterior cul-de-sac, unless the pregnancy be an abdominal one. In the majority of these cases there is a history of sterility for five or six years previous to the abnormal conception.

When the product of conception occupies the fimbriated extremity of the right tube, the points of differentiation are more difficult, owing to the close proximity of the lesion to the position of the appendix and to the negative result of examination per vaginum prior to rupture. Should the two conditions occur coincidently it would be well nigh impossible to differentiate between them.

The chief points to be borne in mind, however, if the two conditions do not cöexist are the history and the absence of inflammatory symptoms prior to the rupture of the extrauterine and the presence of inflammatory symptom in appendicitis.

4. *Suppurating Ovarian Cyst.*—An appendiceal abscess and a small suppurating ovarian cyst on the right side present some symptoms in common which may give rise to difficulties in diagnosis. These symptoms are: painful tumor in the right iliac fossa, which may be made out by vaginal, bimanual and rectal examinations ; symptoms of septicemia ; hectic temperature and history of previous gastric and urinary irritation. The differences, however, are marked and can be distinguished by careful consideration. In ovarian cyst the onset is gradual and a history of infection can generally be elicited. The pain is constant and of a dull character ; by pressure the significant "ovarian pain" may be produced, differing from the colicky and paroxysmal pain of appendicitis, while the tumor itself is more elastic, having apparently thinner walls and a more regular outline.

5. *Ovarian Cyst with Twisted Pedicle.*—Ovarian cyst with a twisted pedicle gives, as a rule, a history of a slowly growing tumor, which is so frequently unaccompanied with pain that its presence has been unsuspected until the accident occurred. The onset of the acute symptoms of a cyst with a twisted pedicle is sudden and is usually caused by excessive peristalsis of the intestines or from sudden changes of the position of the body, causing the tumor to revolve. If the twisting be complete and shut off the circulation the walls of the cyst will rapidly become gangrenous and the patient will become profoundly septic with localised peritonitis, which soon becomes general. Here, again, is seen a resemblance to an attack of appendicitis with abscess formation, but the difference in shape, elasticity, the slow growth, if noted, preceding the sudden onset, the difference in the character of the pain and the tenderness and the lessened degree of rigidity should enable one of experience to distinguish between the two.

If, however, for any reason, it is impossible to make a differential diagnosis, I would advise the lateral incision as the one to be chosen, because appendicitis is so much more common an affection that the chances are in favor of its being the latter. If it is an ovarian cyst the lateral incision can be closed and a median incision made for its removal. The median incision for appendicitis is illogical, unwise and in many instances will present anatomical impossibilities. Where the appendix lies behind the cecum and observes one of the northerly directions, if adherent, will present difficulties in the removal through the ordinary incision. It is, therefore, plain to be seen how much more difficult it would be to attempt its removal through the median line. These difficulties are generally enhanced when there is pus formation and, further, the danger of peritoneal infection is greatly multiplied.

6. *Fibroid Tumor.*—Appendicitis has been confounded with a local inflammation of a portion of the broad ligament overlying an intraligamentary fibroid tumor. The main points in the differential diagnosis are the history of metrorrhagia; the presence of a growth, detected upon vaginal examination, the tenderness and pain which is elicited upon abdominal palpation and is confined to the part of the wall overlying the mass.

7. *A varicose condition of the veins of the broad ligament may simulate chronic appendicitis.*—A differential diagnosis should, however, be easy to make, because on the one hand we have an absence of inflammatory symptoms and a characteristic dull, aching pain with nausea, which disappears when the veins are emptied upon the patient assuming the recumbent position. Again, in chronic appendicitis there is a history of one or more acute attacks with a palpably enlarged organ and the symptoms of chronic inflammation.

8. *Painful menstruation.*—A condition which may be misleading in the differentiation of appendicitis is the sudden onset of pain occurring in young unmarried women of a neurotic temperament at the ushering in of the menstrual period. The onset is sudden, the pain is paroxysmal and accompanied by nausea. There may be more or less rigidity of the lower abdominal walls, which is most frequently bilateral. The presence and the degree of the rigidity of the abdominal walls depends upon the amount of congestion of the ovaries, and whether one or both are involved. The pain, at first paroxysmal, is most severe during the first day of the menstrual flow. After this it may become continuous and, in some instances, last during the entire period. The tenderness and rigidity corresponds to the amount of ovarian congestion. If both ovaries are involved the tenderness will be bilateral. The pain at the onset differs, however, from that of appendicitis, being noninflammatory and localised from the beginning, while in appendicitis there is general abdominal pain, which later becomes localised in the right iliac fossa, while marked intestinal symptoms are present.

*Menopause.*—Some women during the climacteric occasionally complain of symptoms resembling appendicitis. They suffer from localised pain in the right side, gastric and intestinal disturbances and irregular temperature. As absence of the menstrual flow often exists, some difficulty may be experienced in reaching a correct diagnosis, particularly in those cases associated with obesity. A correct diagnosis as to the condition present, however, may be established by careful inquiry into the previous history and by local examination. The latter means will demonstrate the absence of rigidity of the abdominal walls and no palpable enlargement of the appendix. The flushes, backaches and mental symptoms, incident to the menopause, will clear up the diagnosis. The mere mention, however, of the nervous affection, with its ubiquitous symptoms, will suffice.

I met with an unique condition, which I cannot better describe than by relating the state of affairs found at the time of operation. The case, I might state, presented a typical history of acute appendicitis, followed by abscess formation. The appendix was found in relation with the right ovary, to which it was bound by strong adhesions. The ovary was the seat of an abscess which had evidently been infected by the perforated appendix.

### APPENDICITIS AND PREGNANCY.

Pregnancy complicating appendicitis forms one of the most serious conditions which may come under the consideration of the surgeon. Ordinarily appendicitis involves the risk of life; under this condition, however, the life of both mother and child in utero must be taken into consideration. It is claimed by some obstetricians that this is an infrequent occurrence (appendicitis) as a complication of pregnancy. While this may be the case, it is of so great an importance that I believe from its rarity it should receive even more earnest consideration on account of the disastrous results which are likely to follow the formation of an abscess of appendiceal origin at any period near the completion of the term of gestation. When we consider that the gravid uterus with its contents occupies the abdomen to a large extent and that there is a likelihood of the uterus forming a portion of the abscess wall, involving the risk of miscarriage and subsequent shrinking in size of the uterus, the consequent likelihood of adhesions being torn and the escape of the contents of the abscess into the general peritoneal cavity, it is easy to imagine the risk in allowing such a condition of affairs to occur. Women who present the history of previous attacks of appendicitis and complain of local pains and distress in the right iliac fossa, with all the symptoms of chronic appendicitis conjointly with pregnancy, should, in my opinion, be relieved of a chronically diseased appendix to avoid the likelihood of suffering from a condition of affairs as above stated. Although many of them may not go to the formation of pus, still the great uncertainty which hangs over chronic disease of the appendix and the indefinite outlook as to the ultimate outcome when combined with pregnancy, makes it imperative that we should not be conservative enough to endanger the life of the patient.

A question may arise as to the differential diagnosis between peritonitis from a ruptured appendix or ruptured appendiceal abscess and peritonitis resulting from endometritis following abortion, miscarriage or labor at full term. The principal points of differentiation are the history of sudden onset, accompanied by nausea or vomiting, pain, rigidity and tenderness in the case of appendicitis ; while in the latter there is a history of uterine infection prior to the inflammatory invasion of the peritoneal cavity ; there is also an absence early of localised rigidity and tenderness which is diffused in purulent peritonitis.

The rupture of an appendiceal abscess or perforation of the appendix may be followed by a temporary cessation of the more pronounced subjective symptoms previous to general septic peritoneal infection. This may be accompanied by shock.

In puerperal peritonitis the symptoms appear slowly and are progressive and without amelioration.

The symptoms of appendicitis complicating pregnancy are those common to uncomplicated appendicitis, although there may be vomiting throughout the entire period of gestation, not associated with evidences of an inflammatory condition. What is true of appendicitis in general is also true when associated with pregnancy. The likelihood of referred pain, not necessarily confined to the region of the right iliac fossa, is also true when complicated by pregnancy. The diagnosis is rendered more difficult when pregnancy is advanced and the abdomen is filled with the uterus and its contents. The ability to elicit rigidity of the recti muscles is masked on this account, although tenderness will be more pronounced when pus is present. The cases reported of appendicitis complicating pregnancy, I believe, without exception, have given the history of previous attacks of inflammation of the appendix.

Appendicitis, associated with or complicating pregnancy within the early months of gestation, is not as serious by far as when it occurs later in pregnancy. The danger to involvement of the uterine walls in forming a part of the abscess wall, when such is present, is in distinct relation with the elevation of the uterus from the pelvis. The danger of miscarriage also increases as pregnancy advances for the same reason. Changes occur in the placenta when the uterine muscle is included as a part of the abscess wall, especially if the placental attachment corresponds with that portion of the uterine wall forming a portion of the abscess wall. It has been conclusively shown in several instances that degeneration of the placenta has followed abscess formation. In one case the placenta had undergone partial calcarious change; miscarriage followed the evacuation of the abscess. The calcification was noted after the expulsion of the after-birth.

There may be some doubts still in these cases from the fact that the patient has had disturbance of digestion with nausea and vomiting. This condition, however, should not be confounded with nausea and vomiting at the first onset of appendicitis, as this is associated with evidence of inflammation.

A primary acute attack of appendicitis in a female that is pregnant might be mistaken for an approaching miscarriage; in the latter instance, however, the symptoms are not ushered in by severe abdominal pain and, as a rule, there is a lack of nausea attending the onset of local pain.

Acute appendicitis, complicating the later months of pregnancy, is by far a more dangerous condition to deal with. The appendix, if holding any of the southerly or easterly directions, is bound to be in relation with the uterus, and if pus be present the uterus is readily included in the mass of lymph thrown out to wall off the forming pus. Here lies the danger of appendicitis if allowed to go on to pus formation. If removed before pus is formed there is a decreased likelihood of miscarriage, a lessened possibility of peritoneal involvement, adhesion and freedom from peritonitis.

A serious question arises after pus has formed and the uterus is included as a part of the inner wall of the abscess cavity. We must face two dangers: first, the likelihood of infecting the peritoneum primarily; and second, whether it is proper at the time of evacuating the abscess cavity to induce a miscarriage, or allow nature to take its course and run the chance of avoiding an abortion. If we choose the former alternative and evacuate the uterus, after emptying the abscess cavity, it would seem as if the surgeon would be in a position to control the possible infection of the peritoneum by tearing loose of the adhesions to the uterine wall following the contraction of this organ and by a proper disposition of gauze prevent infection of the cavity. If we are to choose the latter alternative and leave the fetus in utero, then there exists a double menace to the future welfare of the patient, a probable infection of the peritoneum, a possible miscarriage or the likelihood of carrying a dead child with the attending risk of uterine infection and the danger of septic endometritis and peritonitis. In addition to the danger of peritoneal infection from the likelihood of the tearing loose of the adhesions to the wall of the uterus, hemorrhage may occur, if the uterus is so bound down that proper contraction of the organ cannot take place.

The salient points which I particularly wish to bring to your attention, I will summarise in the closing paragraphs of this paper.

The many difficulties of diagnosis between disease of the uterine adnexa, pregnancy and appendicitis and the probability of their coëxistence in the same case, brings us face to face with the general rule "that he who opens an abdominal cavity must be prepared to meet any or all the complications which may arise in this most complex portion of the human anatomy."

The first and most important point is the necessity for an early diagnosis, because the good results for operation in appendicitis depend upon the time of operation. The danger increases in direct ratio with the progress of an attack.

In women who are liable to become pregnant and who have had an attack or attacks of appendicitis, the appendix should be taken out in order that the dangerous complication of pregnancy and appendicitis may be avoided.

This is particularly true if an attack of appendicitis supervenes upon pregnancy. The latter condition does not contraindicate operation under the circumstances, but rather, on the other hand, makes operation more imperative.

Another point is one based upon a rich personal experience, covering both operative and nonoperative cases. It is that the number of cases of appendicitis that become perfectly well, cases that do not suffer during the interval between attacks and that are not subject to the certainty of future attacks, are so extremely rare that the question of curative treatment resolves itself to the time of operation.

1634 Walnut Street.

---

If albuminuria complicates pregnancy (Parvin in *Dunglinson's Coll. and Clin. Rec.*) to a serious extent, the patient should be placed upon an almost absolute milk diet, with limewater to aid in its digestion, if necessary. Hot baths should be frequently taken and the bowels should be kept freely open. This complication should be thought of and examinations made for its determination in every case which comes under the physician's care in time to admit of it.

